# An extra pair of eyes: adopting innovative approaches to detect integrity issues in *Naunyn–Schmiedeberg’s Archives of Pharmacology*

**DOI:** 10.1007/s00210-024-03697-1

**Published:** 2025-01-03

**Authors:** Ruben A. van Diest, Roland Seifert, Marcel A. G. van der Heyden

**Affiliations:** 1 Department of Medical Physiology, Division Heart & Lungs, University Medical Center Utrecht, Yalelaan 50, 3584 CM Utrecht, The Netherlands; 2 Institute of Pharmacology, Hannover Medical School, 30625 Hannover, Germany

**Keywords:** Scientific integrity, Scientific fraud, Image issues, Paper mills

## Abstract

Scientific integrity has been increasingly challenged by scientific misconduct and paper mills, resulting in an increase in retractions. *Naunyn–Schmiedeberg’s Archives of Pharmacology* has been significantly impacted by fraudulent submissions, resulting in numerous retractions. By analyzing retraction notes and utilizing a post-publication surveillance strategy, this editorial discusses how this journal continues to deal with problematic publications, uncovers image- and physiological-related integrity issues, and responds to fraudulent activity. By adopting innovative methods to detect integrity issues and transparently communicating our concerns, we aim to increase awareness among scientists and scientific journals.

In recent years, the integrity of scientific research has come under increased scrutiny due to a troubling rise in retractions (Fanelli 2013; Van Noorden 2023; Wittau and Seifert 2024). To motivate retraction measures, journals release retraction notes when they have lost confidence in the accuracy of the published findings, often due to concerns about research integrity. The timing of retractions varies, with retraction notes being released months or even years after the initial publication, depending on when integrity issues are identified, and the investigation process is completed. Common concerns that lead to retractions include data duplication, falsification, and fabrication, with questionable image practices being the most frequently detected type of issue (Byrne et al. 2019; Stroebe et al. 2012).

Importantly, intentional fraudulent activity is not limited to a few rogue authors. In recent years, large-scale operations known as “paper mills” have contributed to the growing problem of falsified and fabricated information in scientific publications (e.g., Byrne and Christopher 2020; Van Noorden 2023). Despite being based on falsified and fabricated data, paper mill products are often difficult to distinguish from authentic research, though they typically share common features, such as commercial email addresses, outsourced research in “service laboratories,” and article structure (Bik 2020; Seifert 2021). Fraudulent activity remains challenging for researchers, peer reviewers, and editors to detect; poses a serious threat to the validity of scientific findings; and can severely damage the careers of the (bystander) researchers involved, harm the broader scientific community, and potentially undermine public trust (Ampollini and Bucchi, 2020).

Despite a thorough review process, integrity issues occasionally arise post-publication in *Naunyn–Schmiedeberg’s Archives of Pharmacology* as well. Since 2000, a total of 42 retraction notes have been released, nearly all issued in the past 5 years (Fig. 1A) (for retraction guidelines, see COPE 2019). A large proportion of these retraction notes were issued in 2019–2021, when the journal experienced an “attack” of fraudulent submissions from paper mills (Seifert 2021). Since then, stricter editorial policies have been in place to protect the integrity of the scientific work and the journal. Some of the policies include an author’s declaration of in-house generated data and requests for institutional email addresses, raw data, and supplemental materials.

**Fig. 1:**
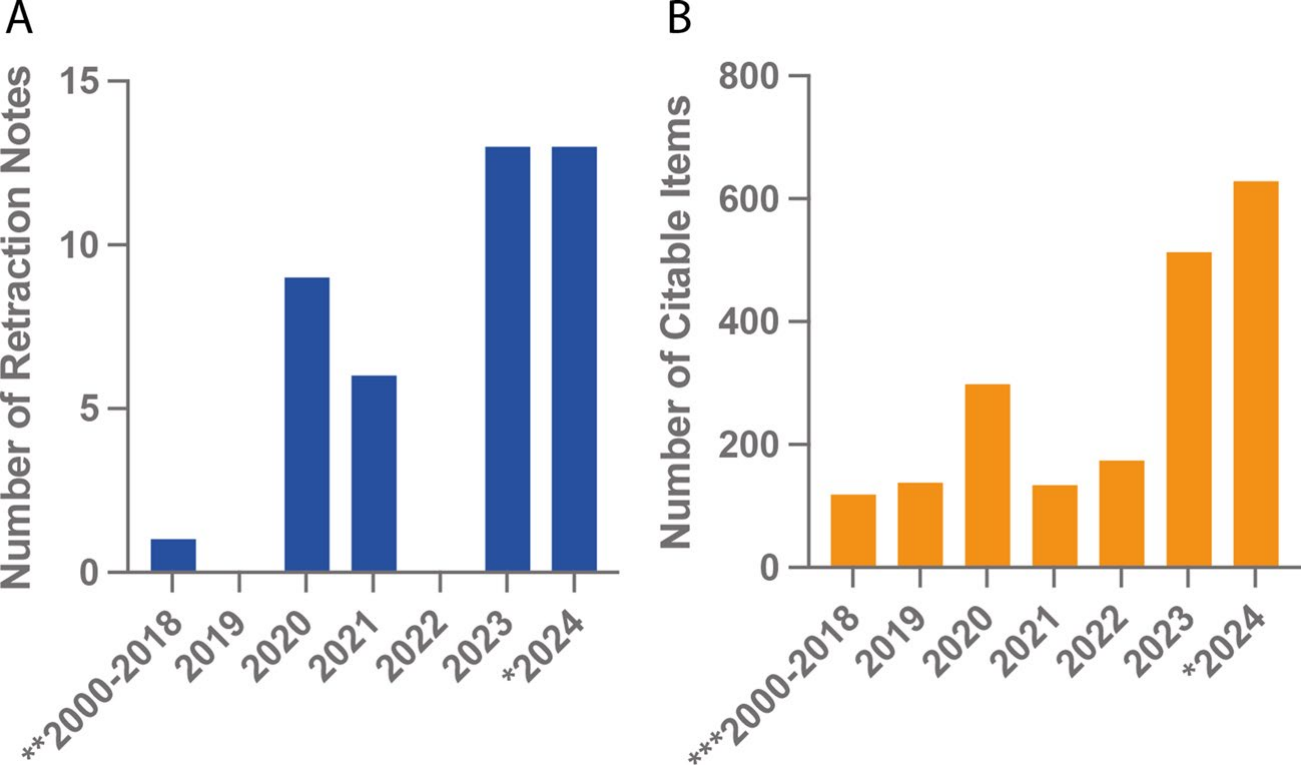
Publications and retraction notes in *Naunyn–Schmiedeberg’s Archives of Pharmacology* since 2000. Barcharts indicate the total number of retraction notes (**A**) or the total number of citable items (**B**) per year. Indicated values are as of November 15, 2024 (*), have been summed up (**), or have been averaged (***). Data is sourced from Springer Nature (Springer Nature) and Clarivate (Clarivate Analytics) per November 2024

Nearly 4 years later, the journal continues to deal with the consequences of fraudulent activities. Since 2000, ~ 4150 citable items have been published with especially notable growth over the past 2 years (Fig. 1B). However, while the number of retractions has increased more modestly relative to the journal’s research output (Fig. 1A and B), the retraction rate, defined as the number of retractions per 10,000 publications, currently stands at 116 per 10,000 publications for the period of 2000–2023. This rate is heavily influenced by retractions since the alleged paper mill “attack” and reflects the journal’s increased awareness and (pro-)active efforts towards responsible science.

To obtain up-to-date insight into the integrity issues and to act accordingly, we critically analyzed retraction notes issued between January 2023 and November 2024 (Tables 1 and 2). During this period, 26 articles have been retracted (please note, retraction notes are cited instead of the retracted articles; Retraction note 2023a, b, c, d, e, f, g, h, i, j, k, l, m, 2024a, b, c, d, e, f, g, h, j, k, l, m). While retraction notes referred to papers published between the years 2001 and 2024, 50.0% (13/26) concerned recently published articles in the 2022–2024 period (Fig. 2). The numbers indicate that problematic publications are an ongoing problem in *Naunyn–Schmiedeberg’s Archives of Pharmacology*.

**Table 1:** Retraction note and author metadata

Retraction note	Publication year of - Original publication - Retraction note	Corresponding author(s) - Geographical location - Email address type - ORCID (Y/N)	Types of concerns - Integrity issue type - #Affected figures
2023a	2017 2023	Egypt Institutional No	Image issues 3
2023b	2023 2023	Egypt Commercial Yes	Image issues 2
2023c	2022 2023	South Korea; Pakistan Institutional; Institutional No; Yes	Image issues 1
2023d	2021 2023	Egypt Institutional Yes	Image issues 3
2023e	2020 2023	Saudi Arabia Institutional Yes	Image issues 2
2023f	2021 2023	Pakistan Institutional Yes	Image issues 1
2023g	2023 2023	Egypt Commercial Yes	Image issues 2
2023h	2019 2023	Egypt Commercial Yes	Image issues 1
2023i	2023 2023	China; China Institutional; Institutional No; No	Image issues 3
2023j	2023 2023	China Commercial No	Ethical violations 0
2023k	2023 2023	India Commercial No	Image issues 4
2023l	2020 2023	Iran Commercial Yes	Image issues 2
2023m	2019 2023	Pakistan; Pakistan Commercial; Institutional No; Yes	Image issues 2
2024a	2022 2024	Pakistan Institutional Yes	Image issues 4
2024b	2023 2024	Turkey Commercial No	Image issues 2
2024c	2018 2024	China Commercial Yes	Image issues 4
2024d	2022 2024	India Commercial Yes	Image issues 1
2024e	2017 2024	Egypt Commercial No	Image issues 2
2024f	2001 2024	Italy Institutional No	Image issues 1
2024g	2022 2024	Poland; India Institutional; Institutional No; No	Bias issues 0
2024h	2023 2024	China Institutional No	Image issues 2
2024i	2022 2024	Pakistan Institutional Yes	Image issues 3
2024j	2013 2024	Egypt Commercial No	Image issues 2
2024k	2021 2024	Egypt Institutional Yes	Image issues 1
2024l	2024 2024	Turkey Institutional Yes	Image issues 1
2024m	2023 2024	China; China Commercial; Institutional No; No	Image issues 1

**Table 2:** Case summaries of retraction notes

Retraction note	Case summary
2023a	The article was retracted due to concerns over image duplication and editing in Fig. 4, Fig. 6, and Fig. 7. The authors provided raw data, however, the same issues were observed. None of the authors agreed to this retraction
2023b	The article was retracted due to concerns over image duplication in Fig. 5c and Fig. 5e and image editing in Fig. 7a-b and d-e. The authors provided raw data, however, the same issues were observed. Two authors did not agree to the retraction, while the remaining authors did not respond to the publisher’s correspondence
2023c	The article was retracted due to concerns over image editing in Fig. 8. The authors provided raw data; however, the same issues were observed. None of the authors responded to the publisher’s correspondence
2023d	The article was retracted due to concerns over image duplication between Fig. 1f and Fig. 5h and between Fig. 6a and 6e. The authors provided raw data and explanations for the issues; however, additional issues were observed. None of the authors agreed to this retraction
2023e	The article was retracted due to concerns over image duplication in Fig. 2b and image editing in Fig. 8. The authors provided raw data; however, discrepancies were observed with the published images. None of the authors responded further to the publisher’s correspondence
2023f	The article was retracted due to concerns over image duplication in Fig. 13. The authors provided raw data; however, the same and additional issues were observed. One author did not agree to the retraction, while the remaining authors did not respond to correspondence of the publisher
2023g	The article was retracted due to concerns over image duplication between Fig. 1 and Fig. 2. Although the authors submitted a corrected figure, they were unable to provide the raw data for validation. One author agreed to the retraction, while the remaining authors did not respond
2023h	The article was retracted due to concerns over image duplication and editing between Fig. 2c and 2f as well as in Fig. 2h. The authors provided raw data; however, the same and additional issues were observed. Two authors did not agree with the retraction, while the remaining authors did not respond to correspondence of the publisher
2023i	The article was retracted due to concerns over image duplications between Fig. 1f and 1g, Fig. 4a and 4b, Fig. 7a and 7b, and Fig. 7c and 7d and image ordering issues in Fig. 4c. All authors agreed to this retraction
2023j	The article was retracted due to ethical violations by the authors. The authors have stated that two authors were unaware of the entire publication. All authors agreed to the retraction; however, two authors disagreed with the retraction wording
2023k	The article was retracted due to concerns over image duplication in Fig. 3 and Fig. 16a and between Fig. 11a and Fig. 15c. The authors provided raw data; however, the same and additional issues were observed. Three authors did not agree with the retraction, while the remaining authors did not respond to correspondence of the publisher
2023l	The article was retracted due to concerns over image duplication and editing within Fig. 3a, 3b, and 3f, as well as image editing in Fig. 7. The authors provided raw data; however, the same and additional issues were observed. One author did not agree with the retraction, while the remaining authors did not respond to the publisher’s correspondence
2023m	The article was retracted due to concerns over image duplication in Fig. 10a and image duplication and editing in Fig. 8b. None of the authors responded to the publisher’s correspondence
2024a	The article was retracted due to concerns over image duplication in Fig. 10 and Fig. 11 and over image duplication and editing within Fig. 2d and Fig. 3. The authors provided raw data; however, the same and additional issues were observed. None of the authors responded further to the publisher’s correspondence
2024b	The article was retracted due to concerns over image duplication within Fig. 3 and Fig. 5c. It is not explicitly stated whether the authors provided the raw data. Four authors did not agree to the retraction, while the remaining author did not respond to correspondence of the publisher
2024c	The article was retracted due to concerns over image duplication with previously published articles in Fig. 2a, Fig. 3, and Fig. 6a. It is not explicitly stated whether the authors provided the raw data; however, additional issues were reported by the authors for Fig. 4a. None of the authors responded to further correspondence from the publisher
2024d	The article was retracted due to concerns over image duplication and editing within Fig. 11a-d. It is not explicitly stated whether the authors provided the raw data; however, the authors have stated that Fig. 11b-c were edited. None of the authors agreed to this retraction
2024e	The article was retracted due to concerns over image duplication in Fig. 2a and 2g and image editing in Fig. 5b-d and 5 g. The authors provided raw data; however, the same and additional issues were observed. One author did not agree to the retraction, while the remaining authors did not respond to correspondence of the publisher
2024f	The article was retracted due to concerns over image duplication and editing in Fig. 1 with a previously published article. It is not explicitly stated whether the authors provided the raw data; however, the authors have provided an explanation for the issues, which was considered insufficient by the publisher. Three authors did not agree to the retraction, while the remaining authors did not respond to correspondence of the publisher
2024g	The article was retracted due to concerns over bias or lack of balance issues. None of the authors agreed to this retraction
2024h	The article was retracted due to image duplications in Fig. 2 and in Fig. 5e. Although the authors provided raw data, the same issues were observed. All authors disagreed with this retraction
2024i	The article was retracted due to concerns over image duplication and editing in Fig. 5, Fig. 6, and Fig. 7 and image duplication and editing in Fig. 5 with a previously published article. The authors provided raw data; however, the same and additional issues were observed. None of the authors responded to further correspondence from the publisher
2024j	The article was retracted due to concerns over image duplication between Fig. 4 and Fig. 5. The authors provided raw data, however, the same issues were observed. One author did not agree to the retraction, while the remaining authors did not respond to correspondence of the publisher
2024k	The article was retracted due to concerns over image duplication within Fig. 3b. The authors were unable to provide the raw data for validation. Three authors did not agree to the retraction, while the remaining author did not respond to correspondence of the publisher
2024l	The article was retracted due to concerns over image duplication within Fig. 4. The authors were unable to provide the raw data for validation. Four authors agreed to the retraction, while the remaining authors did not respond to correspondence of the publisher
2024m	The article was retracted due to concerns over image duplication within Fig. 2a. The authors were unable to provide the raw data for validation. One author agreed and one author disagreed to the retraction, while the remaining authors did not respond to correspondence of the publisher

**Fig. 2:**
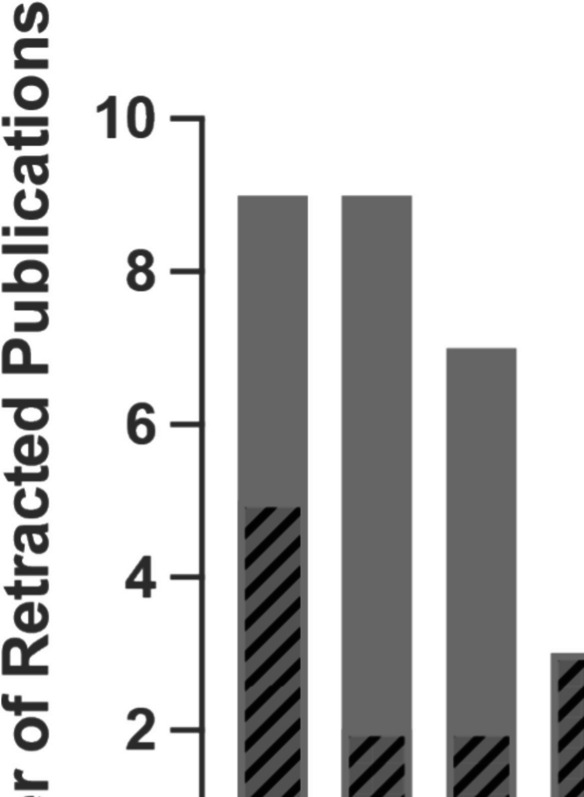
Retracted articles in *Naunyn–Schmiedeberg’s Archives of Pharmacology* since 2000. Barchart indicates the number of retracted articles, issued in 2000–2024 (solid) or in 2023–2024 (patterned), categorized by the year of publication of the original paper. Indicated values are as of November 15, 2024 (*) or have been summed up (**). Data is sourced from Springer Nature (Springer Nature) per November 2024

Looking critically at the author metadata of the retraction notes (Table 1), we observed that corresponding authors were geographically located in Egypt (8/31), China (7/31), Pakistan (6/31), India (3/31), Turkey (2/31), Iran (1/31), South Korea (1/31), Saudi Arabia (1/31), Poland (1/31), and Italy (1/31). Some of these countries have been linked to relatively higher retraction rates (Van Noorden 2023). In line with the journal’s stricter editorial policies (Seifert 2021), we observed that 58.1% (18/31) of corresponding authors provided institutional email addresses, and that 64.5% (20/31) included ORCIDs. While these measures show progress towards more transparent authorship, they are not yet uniformly adopted worldwide. Additionally, as we are aware of the phenomenon of serial cases of fraud (Samp et al. 2012; Van der Heyden 2024), we checked for patterns of serial retractions. We found two cases of serial retractions that concerned 34.6% (9/26) of the retracted articles (Retraction note 2023c, 2023f, 2023m, 2024a and 2023a, 2023d, 2023h, 2024e, 2024k). To which extent the authors associated with these retractions were involved in retractions outside *Naunyn–Schmiedeberg’s Archives of Pharmacology* has not been investigated.

We also analyzed the authors’ communication with the journal and the types of concerns raised, as described in the retraction notes. Of the 26 retractions analyzed, 96.2% (25/26) of the author groups responded to the publisher’s correspondence, with most offering raw data and/or explanations (Table 2). However, as the articles became retracted subsequently, these responses did not fully address the journal’s worries about the integrity of the publications. Alarmingly, only 37.6% (65/173) of individual authors responded to the retraction notes, which raises concerns about the lack of engagement from a significant portion of the authors. Additionally, 92.3% (24/26) of the publications were retracted due to image issues (Table 1), some of which were reported to the journal directly and/or (anonymously) in Pubpeer (Pubpeer). According to the retraction notes, a total of 50 figures were identified as problematic, covering various experimental techniques and published materials (Fig. 3A). While the majority of cases involved (partial) image duplication, some cases of advanced editing were observed (Fig. 3B). While we acknowledge that most of these issues look clumsy, these and most definitely more advanced manipulations still pass extensive review processes. The remaining two retractions were associated with ethical concerns or bias issues.

**Fig. 3:**
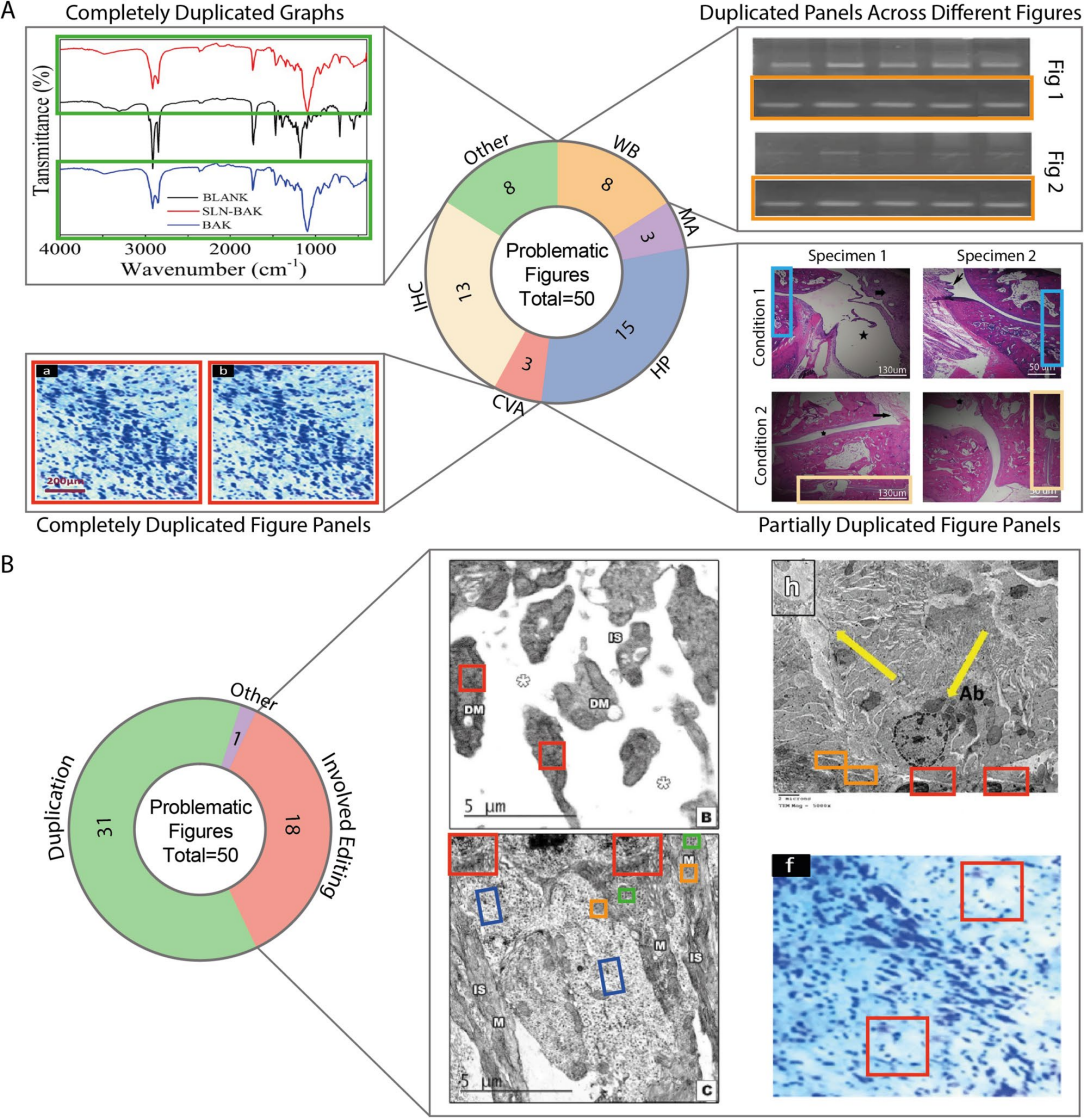
Donut charts presenting the number of problematic figures in the retracted articles, categorized by experimental technique or graph (**A**) or by type of problem (**B**) surrounded by examples images from retracted articles as specified in the corresponding retraction notes. Please note, retraction notes are cited instead of retracted articles. **A** Excerpts of Fig. 3 (Retraction note 2023l), Fig. 4–5 (Retraction note 2024j), Fig. 3 (Retraction note 2023k), and Fig. 7 (Retraction note 2024i). Data originates from the same Nissl staining, western blot, Fourier transform infrared spectroscopy, and histopathological samples, respectively, but is presented as different experimental conditions. **B** Excerpts of Fig. 8 (Retraction note 2023e), Fig. 2 (Retraction note 2023h), and Fig. 3 (Retraction note 2023l). Data is presented as electron microscopy and Nissl staining samples, respectively, but contains repeated patterns. Duplicated areas are highlighted in colored rectangles (yellow arrows in the electron microscopy image of **B** are by the original authors). Adapted with permission of the Research Integrity Group of *Naunyn–Schmiedeberg’s Archives of Pharmacology*. WB, western blot; MA, morphological assessment; HP, histopathology; IHC, immunohistochemistry; CVA, cell viability assay

In addition to analyzing the retraction notes, we have, since February 2024, performed post-publication screening of recently published *Naunyn–Schmiedeberg’s Archives of Pharmacology* articles on a daily to weekly basis, following a methodology described previously (Van der Heyden 2021). To our concern, we regularly identified image irregularities, including extreme cases, and physiological improbabilities, such as unusually large rodents, e.g., mice of approximately 400 g, and other questionable experimental parameters and/or materials. We believe these issues may stem from the limitations of Artificial Intelligence (AI) and/or paper mills in accurately valuing parameters in rodent studies. However, as AI technology continues to advance, we anticipate that these issues will become less apparent in the coming years. Since the start of our screening activities, we flagged several recently published papers most of which contained one or more figure issues, and these are currently under investigation, corrected, or retracted (Retraction note 2024l, 2024m). And to become better aware of serial fraud cases, additional publications of the same author group in *Naunyn–Schmiedeberg’s Archives of Pharmacology* also become subject to our screening process. In addition to these measures, the journal will also be introducing new AI tools, known as Geppetto and Snappshot, to detect figure issues and AI-generated fake content (Welschot 2024; Springer Nature Press Release 2024).

To conclude, despite the challenges posed by paper mills and other integrity issues, *Naunyn–Schmiedeberg’s Archives of Pharmacology* remains committed to publishing high-quality, reliable research output. In response to these challenges, the journal has already implemented stricter editorial policies and enhanced post-publication surveillance, which will soon be supported by advanced AI tools to ensure the integrity of published work. We strongly urge authors to respect the highest level of research integrity to serve science instead of personal gain. We also urge evaluation committees worldwide to respect this attitude towards good science.
